# αβ-T cell receptor transduction gives superior mitochondrial function to γδ-T cells with promising persistence

**DOI:** 10.1016/j.isci.2023.107802

**Published:** 2023-08-31

**Authors:** Mikiya Ishihara, Hiroshi Miwa, Hiroshi Fujiwara, Yasushi Akahori, Takuma Kato, Yoshimasa Tanaka, Isao Tawara, Hiroshi Shiku

**Affiliations:** 1 Department of Medical Oncology, Mie University Hospital, 2-174 Edobashi, Tsu, Mie 514-8507, Japan; 2 Department of Personalized Cancer Immunotherapy, Mie University Graduate School of Medicine, 1577 Kurimamachiya-cho, Tsu, Mie 514-8507, Japan; 3 Department of Cellular and Molecular Immunology, Mie University Graduate School of Medicine, Tsu, Mie 514-8507, Japan; 4 Center for Medical Innovation, Nagasaki University, 1-7-1 Sakamoto, Nagasaki 852-8588, Japan; 5 Department of Hematology and Oncology, Mie University Graduate School of Medicine, 2-174 Edobashi, Tsu, Mie 514-8507, Japan

**Keywords:** Cellular therapy, Molecular biology, Immunology

## Abstract

Adoptive cell therapy using allogeneic γδ-T cells is a promising option for off-the-shelf T cell products with a low risk of graft-versus-host disease (GVHD). Long-term persistence may boost the clinical development of γδ-T cell products. In this study, we found that genetically modified Vγ9^+^Vδ2^+^ T cells expressing a tumor antigen-specific αβ-TCR and CD8 coreceptor (GMC) showed target-specific killing and excellent persistence. To determine the mechanisms underlying these promising effects, we investigated metabolic characteristics. Cytokine secretion by γδ-TCR-stimulated nongene-modified γδ-T cells (NGMCs) and αβ-TCR-stimulated GMCs was equally suppressed by a glycolysis inhibitor, although the cytokine secretion of αβ-TCR-stimulated GMCs was more strongly inhibited by ATP synthase inhibitors than that of γδ-TCR-stimulated NGMCs. Metabolomic and transcriptomic analyses, flow cytometry analysis using mitochondria-labeling dyes and extracellular flux analysis consistently suggest that αβ-TCR-transduced γδ-T cells acquire superior mitochondrial function.

In conclusion, αβ-TCR-transduced γδ-T cells acquire superior mitochondrial function with promising persistence.

## Highlights

• Long-term persistence may boost the clinical development of γδ-T cell products
• αβ-TCR transduction into γδ-T cells showed promising persistence
• αβ-TCR transduced γδ-T cells could utilize more mitochondrial energy metabolism

## Graphical abstract

**Figure:**
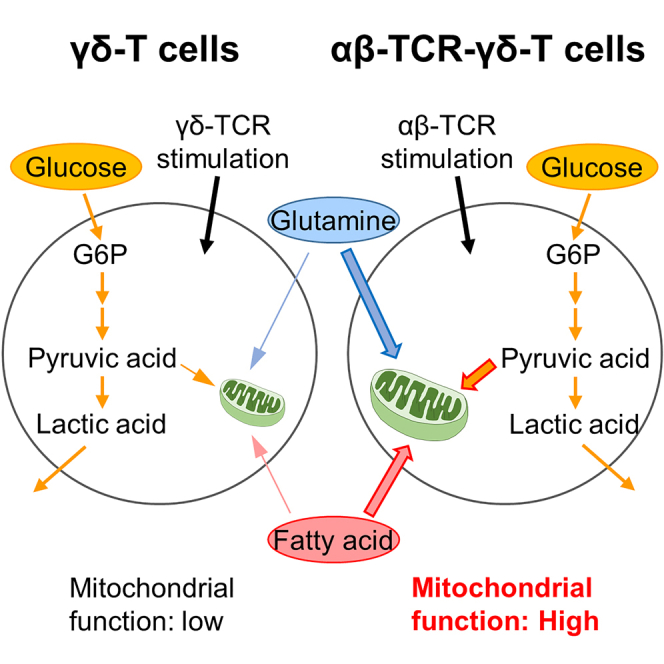

## Introduction

A new era of immunotherapy for cancer has arrived with the application of immune checkpoint inhibitors and adoptive cell therapy with genetically modified T cells directed against cancer antigens. Chimeric antigen receptor-engineered T cells (CAR-T cells) targeting CD19 have shown clinical benefits in clinical trials^1^^,^^2^^,^^3^ and are an available standard therapy for B cell malignancies. T cell receptor (TCR)-engineered T cells (TCR-T cells) are less developed than CAR-T cells, but no conclusion has been reached as to whether CAR-T cells or TCR-T cells are superior. One of the characteristics of TCR-T cells is their ability to target intracellularly expressed antigens. New York esophageal squamous cell carcinoma 1 (NY-ESO-1) is an intracellularly expressed cancer-testis antigen with a wide range of expression in tumors but a limited range of expression in normal tissues.^4^^,^^5^^,^^6^ NY-ESO-1 is the most studied antigen in TCR-T cell clinical trials.^7^ Our group conducted clinical trials of TCR-T cells targeting NY-ESO-1-expressing tumors^8^^,^^9^ using the G50A + A51E TCR, an NY-ESO-1-specific TCR recognizing the NY-ESO-1_p157-165_ peptide (NE1_p157_) and HLA-A∗02:01 complex. This TCR had two amino acid replacements in the CDR2 region of the TCRβ chain to produce high affinity,^10^ which differs from the one reported by Robbins et al.^11^^,^^12^^,^^13^ G50A + A51E TCR-T cells showed tolerable toxicity and promising efficacy.^8^^,^^9^

Despite the successes described previously, there are two main issues with adoptive T cell therapy using αβ-T cells: mispairing of endogenous and engineered αβ-TCRs^14^ and difficulties in using allogeneic αβ-T cells for rapid and chemotherapy-damage-free cell product preparation due to graft-versus-host disease (GVHD). To address these issues, we focused on γδ-T cells. αβ-TCR-transduced γδ-T cells targeting cancer antigens have already been studied.^15^^,^^16^^,^^17^ γδ-T cells recognize tumors in an MHC-independent manner^18^^,^^19^ and have no risk of mispairing endogenous and engineered αβ-TCRs. Tumor infiltration by γδ-T cells is a good prognostic marker for many cancers.^20^ Although adoptive cell transfer of nongene-modified γδ-T cells (NGMCs) has been studied,^21^^,^^22^^,^^23^^,^^24^^,^^25^ these cells have not achieved a remarkable improvement in survival. Redirection of γδ-T cells by αβ-TCR gene transduction may yield antitumor activities against NGMC-resistant tumors and resolve the two aforementioned issues with αβ-T cell products.

In this study, we evaluated genetically modified Vγ9^+^Vδ2^+^ T cells expressing NY-ESO-1-specific αβ-TCR and the CD8 coreceptor (NE1-GMCs), with a specific focus on their metabolic preferences in association with their effector function and persistence.

## Results

### Characteristics of αβ-TCR-transduced γδ-T cells

**Figure 1:**
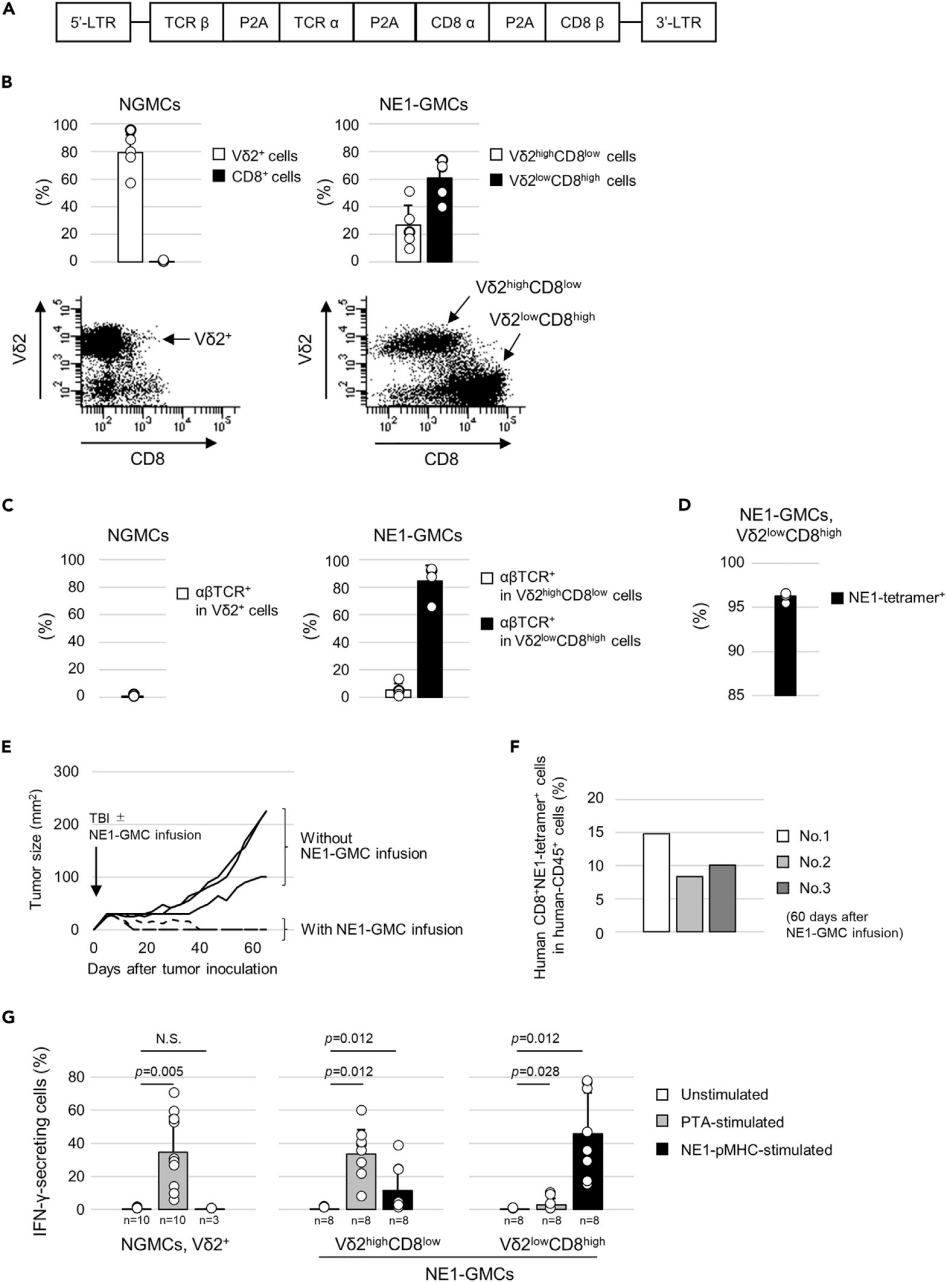
Characteristics of γδ-T cells in this study (A) Construction of a retroviral vector to transduce the NY-ESO-1 TCR used in this study. (B) Mean frequency (upper) and representative data (lower) of Vδ2 and CD8 expression in nongene-modified T cells (NGMCs, left, n = 5) and NY-ESO-1-TCR gene-modified T cells (NE1-GMCs, right, n = 5). Dot plots show representative data of the expression of Vδ2 and CD8 in NGMCs (left) and NE1-GMCs (right). (C) Mean frequencies of αβTCR^+^ cells in NGMC Vδ2^+^ (left, n = 4), NE1-GMC Vδ2^high^CD8^low^ and NE1-GMC Vδ2^low^CD8^high^ cells (right, n = 4). (D) Mean frequency of NE1-tetramer-positive cells in NE1-GMC Vδ2^low^CD8^high^ cells (n = 3). The error bar shows the standard deviation. E) Tumor size change in NW-MEL-38 melanoma cell (HLA-A2^+^, NY-ESO-1^+^)-bearing NOG mice. Mice were treated with total body irradiation (TBI) (2 Gy) and then infused with (dotted lines, n = 3) or without (solid lines, n = 3) NE1-GMCs. The frequency of CD8^+^NE1-Tetramer^+^ cells in infused NE1-GMCs was 62.2%. (F) Frequency of human CD8^+^NE1-tetramer^+^ cells in human CD45^+^ cells. Sixty days after NE1-GMC infusion, peripheral blood was obtained from 3 mice, as shown in Figure 2A, and examined. (G) Frequencies of IFN-γ-secreting NGMC Vδ2^+^, NE1-GMC Vδ2^high^CD8^low^ and NE1-GMC Vδ2^low^CD8^high^ cells: no stimulation (white), stimulated with PTA (gray), or NE1-pMHC (black).

γδ-T cells induced by a novel bisphosphonate prodrug, tetrakis-pivaloyloxymethyl 2-(thiazole-2-ylamino) ethylidene-1,1-bisphosphonate (PTA),^26^ exhibited a Vγ9^+^Vδ2^+^ phenotype (data not shown). Since coexpression of CD8αβ enhanced the antitumor effect of NY-ESO-1-specific αβ-TCR (G50A + A51E TCR)-transduced γδ-T cells (Figures S1A–S1D), we produced a retroviral vector encoding the G50A + A51E TCR gene and the human CD8αβ gene and used it in this study (Figure 1A). In NE1-GMCs, there were two distinct populations: Vδ2^high^CD8^low^ and Vδ2^low^CD8^high^ cells (Figure 1B). Transduced NY-ESO-1-specific αβ-TCR was mainly observed in NE1-GMC Vδ2^low^CD8^high^ cells (Figures 1C and 1D), and there was a positive correlation between NY-ESO-1-specific αβ-TCR and CD8 expression (Figure S2A). Transduction of αβ-TCR had no obvious effect on the γδ-T cell phenotype, TOX expression and cytokine production (Figures S2B–S2D). NE1-GMCs tended to have lower PD-1 expression (Figure S2C). NE1-GMCs induced antigen-specific apoptosis in NE1_p157_-pulsed target cells and NY-ESO-1^+^HLA-A2^+^ tumor cells (Figures S3A–S3C). To assess *in vivo* persistence, we used NY-ESO-1-expressing tumor-bearing mice. NY-ESO-1^+^HLA-A2^+^ tumors regressed after NE1-GMC transfer with total-body irradiation (Figure 1E). NE1-GMCs were observed in the peripheral blood of mice 60 days after infusion (Figures 1F and S3D). In the control group, observation was terminated 65 days after tumor inoculation because of tumor growth. In contrast, in the NE1-GMC-infused group, no tumor growth was observed 90 days after tumor inoculation, and there was no tumor growth after reinoculation of the tumor. Vδ2^high^CD8^low^ NE1-GMCs secrete IFN-γ in response to PTA. In contrast, Vδ2^low^CD8^high^ NE1-GMCs secreted IFN-γ in response to NE1-pMHC (Figure 1G).

### Effector function and metabolism dependency of IFN-γ secretion in NE1-GMCs

In cultures without cytokine supplementation, cell growth of PTA-treated γδ-T cells was suppressed (Figures S4A and S4B). PTA-treated NGMCs functioned as antigen-presenting cells, and the possibility of mutual cytotoxicity could not be ruled out (Figure S4C). In contrast, compared to NE1-GMCs, TCRγδ-specific agonistic mAb (clone IMMU510)-stimulated NGMCs remained at a similar frequency (Figure S4A) and showed similar cell proliferation (Figure S4D). For the aforementioned reasons, IMMU510 was used to stimulate NGMCs in subsequent experiments.

**Figure 2:**
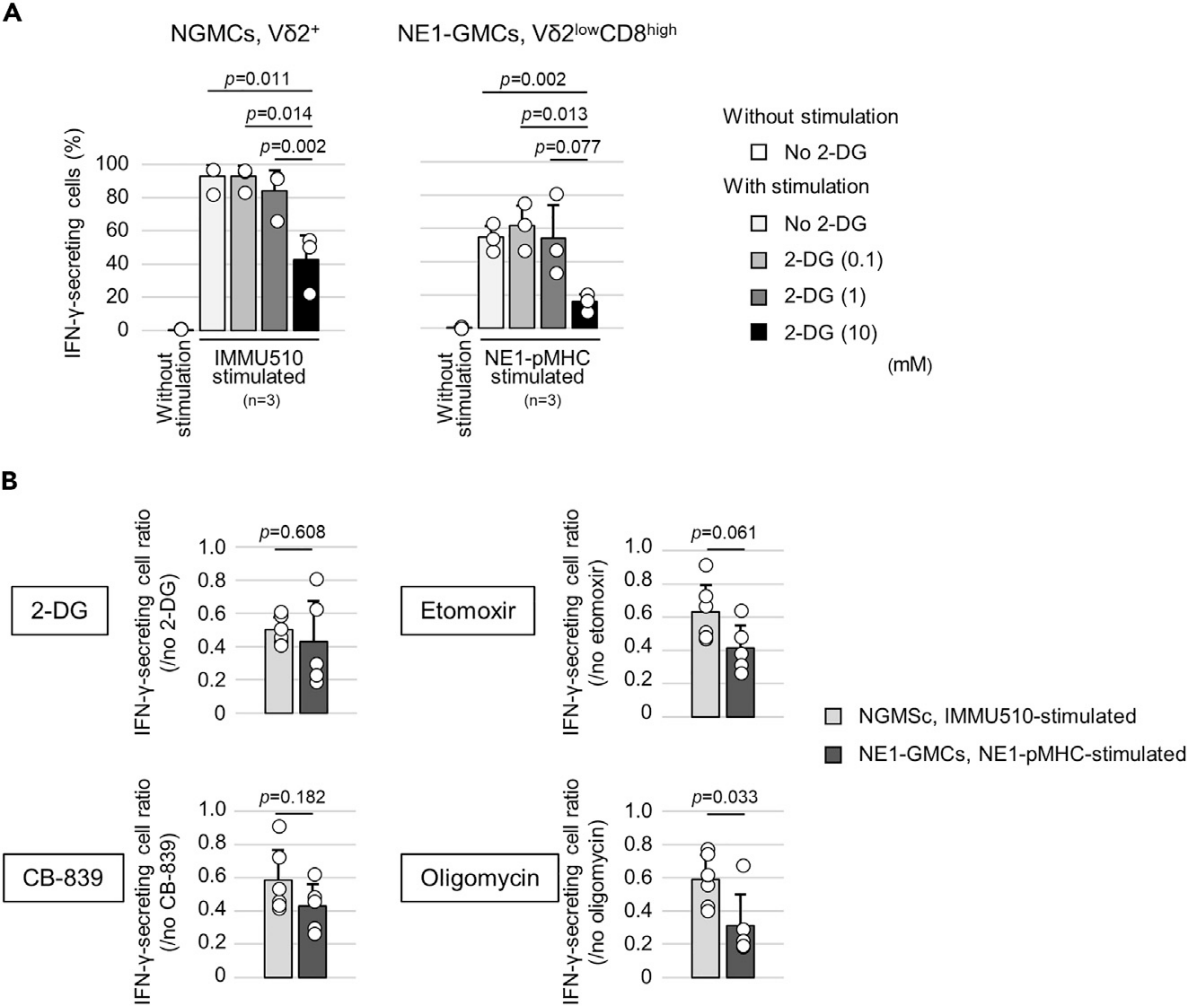
NY-ESO-1-specific response and persistence of NE1-GMCs and comparison of the effects of metabolic inhibition on IFN-γ secretion between NGMCs and NE1-GMCs (A) Frequencies of IFN-γ-secreting cells in NGMC Vδ2^+^ cells and NE1-GMC Vδ2^low^CD8^high^ cells in media with different 2-DG concentrations. The means of 3 experiments using γδ-T cells induced from 2 healthy donors are shown in the bar graph. (B) Frequencies of IFN-γ-secreting cell ratios determined by comparing IMMU510-stimulated NGMC Vδ2^+^ cells (6 experiments using γδ-T cells induced from 3 healthy donors) and NE1-pMHC-stimulated NE1-GMC Vδ2^low^CD8^high^ cells (6 experiments using γδ-T cells induced from 3 healthy donors). The concentrations of metabolic inhibitors were 2-DG (10 mM), etomoxir (100 μM), CB-839 (2 μM), or oligomycin (2 μg/mL). The error bar shows the standard deviation.

To assess the dependence on glucose metabolism, examinations of cytokine secretion by γδ-T cells using metabolic inhibitors were performed. IFN-γ secretion by NGMCs and NE1-GMCs decreased with increasing concentrations of 2-deoxy-D-glucose (2-DG), a glycolysis inhibitor (Figure 2A). Compared with that of IMMU510-stimulated NGMCs, the IFN-γ secretion of NE1-pMHC-stimulated NE1-GMCs was more strongly suppressed by oligomycin (an ATP synthase inhibitor) (Figure 2B and Table S1). The same trend of inhibition of IFN-γ secretion by oligomycin was observed for p40Tax-pMHC-stimulated p40Tax-specific αβ-TCR^27^ and CD8-transduced GMCs (p40Tax-GMCs) (Figure S5). Etomoxir (a fatty acid oxidation inhibitor) and CB-839 (a glutaminase inhibitor) also tended to inhibit IFN-γ secretion more in αβTCR-transduced GMCs, suggesting that αβTCR-transduced GMCs utilize more mitochondrial functions.

### Metabolomic analysis of NGMCs and NE1-GMCs

**Figure 3:**
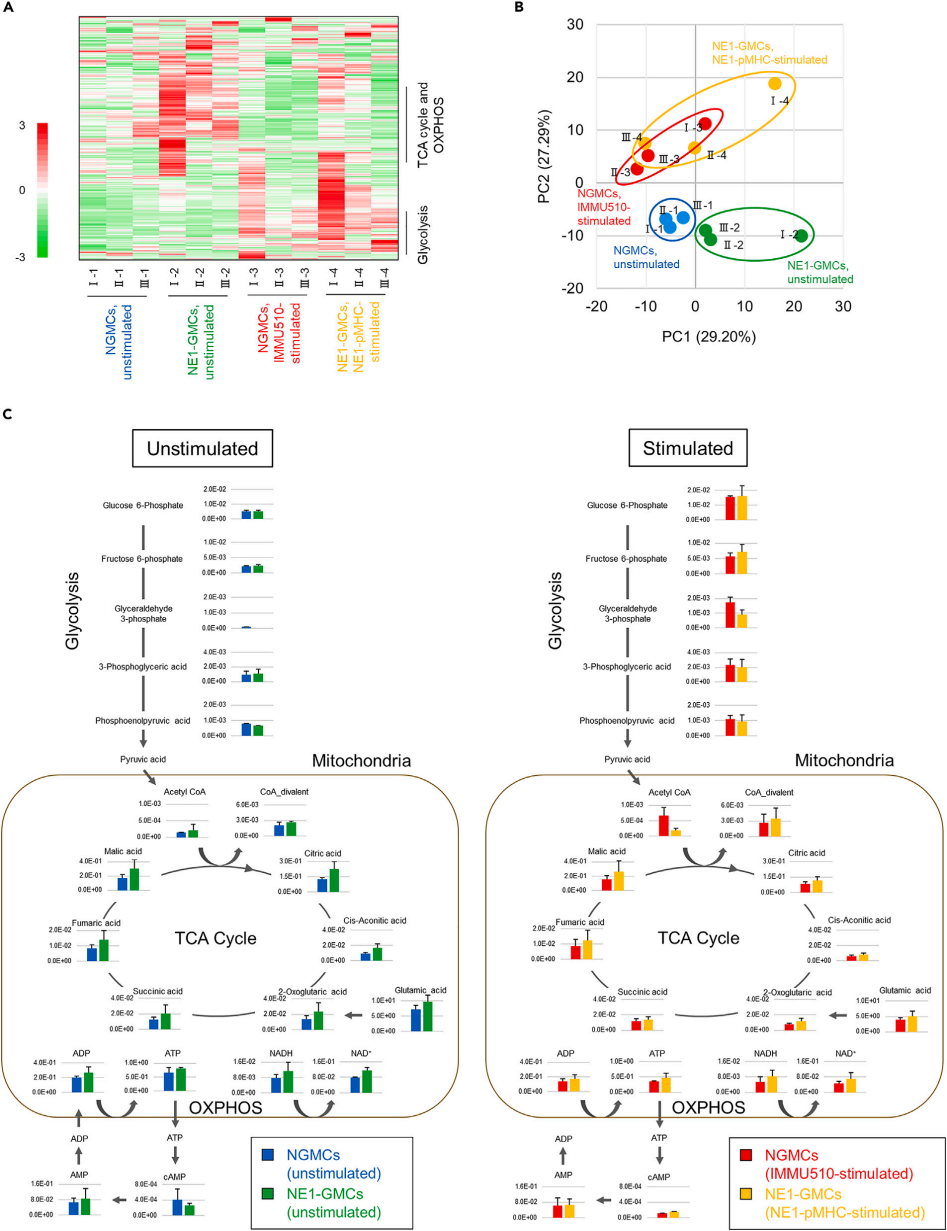
Metabolomic analysis of NGMCs and NE1-GMCs Three samples from three different healthy donors per group were assessed. The Roman numerals (I, II, and III) at the top of each sample name indicate that the T cells were derived from the same healthy donor and induced on the same day. (A) Heatmap of 357 metabolites (cationic:195, anionic:162) detected by CE-FTMS compared among unstimulated NGMCs (blue; Ⅰ-1, Ⅱ-1, and Ⅲ-1), unstimulated NE1-GMCs (green; Ⅰ-2, Ⅱ-2, and Ⅲ-2), IMMU510-stimulated NGMCs (red; Ⅰ-3, Ⅱ-3, and Ⅲ-3), and NE1-pMHC-stimulated NE1-GMCs (orange; Ⅰ-4, Ⅱ-4, and Ⅲ-4). The bars on the right show the range of metabolites involved in glycolysis (lower panel) and the TCA cycle and OXPHOS (upper panel). (B) Principal component analysis of 357 detected metabolites. Each circle indicates unstimulated NGMCs (blue), unstimulated NE1-GMCs (green), IMMU510-stimulated NGMCs (red), and NE1-pMHC-stimulated NE1-GMCs (orange). (C) Bar graph of the metabolites involved in glycolysis, the TCA cycle, and OXPHOS in unstimulated and stimulated states. Each bar represents the mean relative areas of the indicated metabolites. Error bars indicate the standard deviation.

Metabolites from unstimulated and stimulated NGMCs and NE1-GMCs were analyzed using a combination of capillary electrophoresis and Fourier transform mass spectrometry (CE-FTMS), and 357 metabolites (cationic:195, anionic:162) were detected (Figure 3A). Metabolites involved in the TCA cycle and OXPHOS were strongly correlated with PC1, which separated unstimulated NE1-GMCs from unstimulated NGMCs (Figure 3B and Table S2). NE1-GMCs contained more metabolites of the tricarboxylic acid (TCA) cycle and oxidative phosphorylation (OXPHOS) in both unstimulated and stimulated states (Figure 3C). The frequencies of Vδ2^+^ cells in NGMCs and CD8^+^ cells in NE1-GMCs used in the study were similar (Figures S6A and S6B).

### Transcriptomic analysis

**Figure 4:**
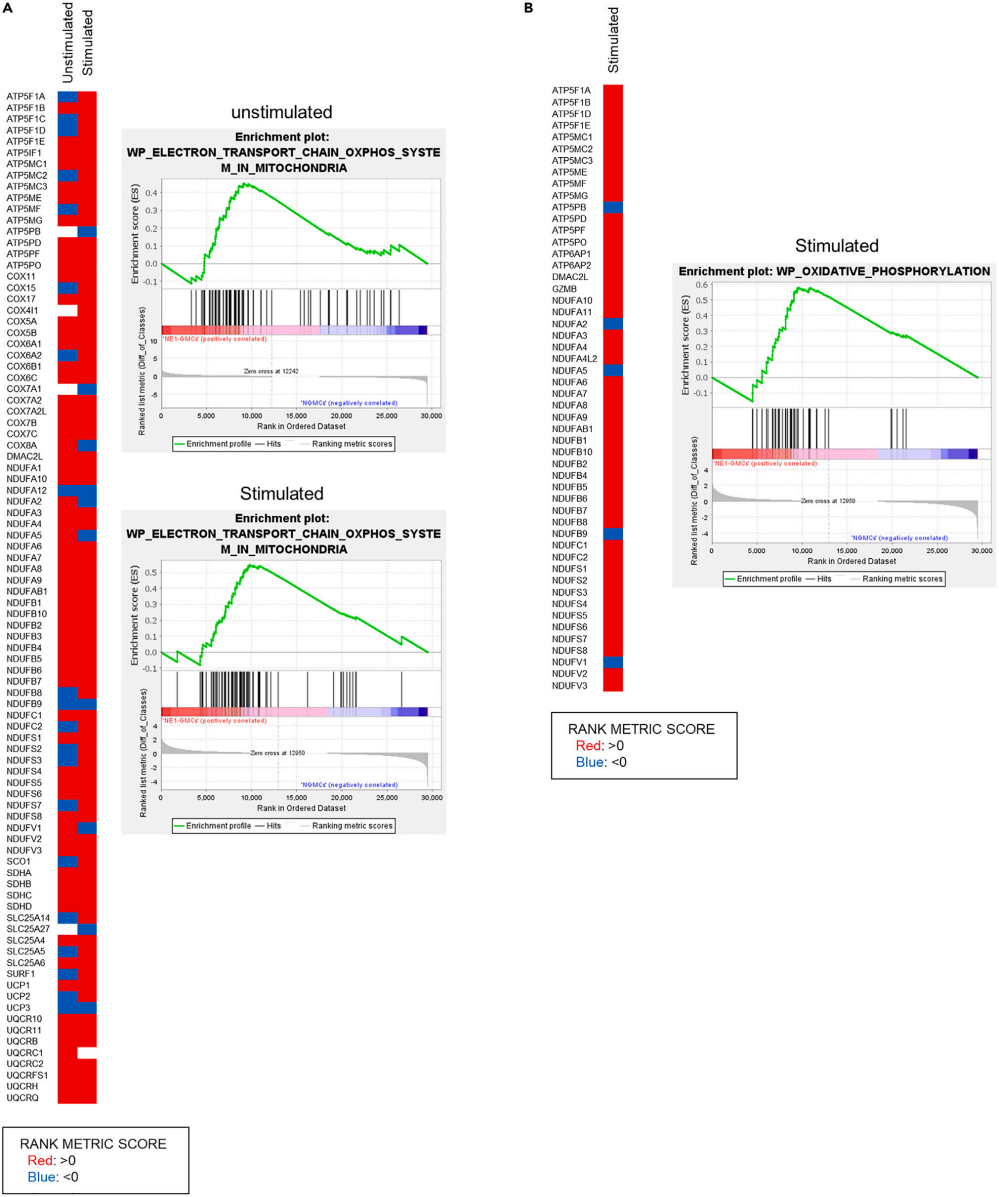
Transcriptomic analysis of NGMCs and NE1-GMCs Gene set enrichment analysis (GSEA) between NE1-GMCs purified with CD8 microbeads and NGMCs under unstimulated and stimulated conditions was performed. (A) Heatmap of rank metric scores and plots of the enrichment scores for the “ WP electron transport chain: oxphos system in mitochondria” gene set in the unstimulated and TCR-stimulated states are shown. (B) Heatmap of rank metric scores and plots of the enrichment scores for the “WP oxidative phosphorylation” gene set in the TCR-stimulated state are shown. The “unstimulated” rank metric score shows unstimulated NE1-GMCs versus unstimulated NGMCs, and the “stimulated” rank metric score shows NE1-pMHC-stimulated NE1-GMCs versus IMMU510-stimulated NGMCs. Rank metric score >0 (red) means higher in NE1-GMCs, <0 (blue) means lower in NE1-GMCs, compared with NGMCs. One sample per group was analyzed.

RNA microarray analysis was performed using cells from the four groups as in the metabolomic analysis. Cells were newly induced on different days from those used for metabolomic analysis. Gene set enrichment analysis (GSEA) showed a trend toward increased expression of genes related to mitochondria in NE1-GMCs compared to NGMCs. Gene expression associated with the “electron transport chain: oxphos system in mitochondria” of WikiPathways was higher in NE1-GMCs than in NGMCs under both unstimulated and stimulated conditions (Figure 4A and Table 1). Gene expression associated with the “oxidative phosphorylation” of WikiPathways was higher in NE1-GMCs than in NGMCs in the stimulated state (Figures 4B and Table 1).

**Table 1:** Summary of gene set enrichment analysis (GSEA): detected mitochondrial metabolism-related gene sets

Gene set	SIZE	Unstimulated NE1-GMCs per unstimulated NGMCs				NE1-pMHC-stimulated NE1-GMCs per IMMU510-stimulated NGMCs			
		ES	NES	NOM p value	FDR q-value	ES	NES	NOM p value	FDR q-value
WP_MITOCHONDRIAL_COMPLEX_III_ASSEMBLY	15	0.69	1.44	0.057	1.000	0.53	1.07	0.401	1.000
WP_ELECTRON_TRANSPORT_CHAIN_OXPHOS_SYSTEM_IN_MITOCHONDRIA	90	0.45	1.33	0.042	1.000	0.55	1.57	0.004	1.000
WP_GLYCOGEN_SYNTHESIS_AND_DEGRADATION	40	0.50	1.30	0.109	1.000	0.52	1.31	0.117	1.000
WP_TCA_CYCLE_AND_DEFICIENCY_OF_PYRUVATE_DEHYDROGENASE_COMPLEX_PDHC	16	0.61	1.29	0.153	1.000	0.66	1.36	0.113	1.000
WP_MITOCHONDRIAL_COMPLEX_I_ASSEMBLY_MODEL_OXPHOS_SYSTEM	49	0.46	1.23	0.134	1.000	0.38	1.00	0.401	1.000
WP_OXIDATIVE_PHOSPHORYLATION	52	0.42	1.13	0.230	1.000	0.58	1.54	0.006	1.000
WP_MITOCHONDRIAL_COMPLEX_IV_ASSEMBLY	31	0.47	1.13	0.272	1.000	0.55	1.31	0.112	1.000
WP_TCA_CYCLE_AKA_KREBS_OR_CITRIC_ACID_CYCLE	18	0.50	1.09	0.346	1.000	0.57	1.22	0.194	1.000

ES, Enrichment Score; FDR, False discovery rate; NE1-GMCs, genetically modified γδ-T cells expressing an NY-ESO-1-specific αβ-TCR and the CD8 coreceptor; NES, Normalized Enrichment Score; NE1-pMHC, NY-ESO-1_p157-165_ peptide and HLA-A∗02:01 complex; NGMCs, non-gene-modified γδ-T cells; NOM, Nominal.

### Mitochondrial phenotype assessed by flow cytometry

**Figure 5:**
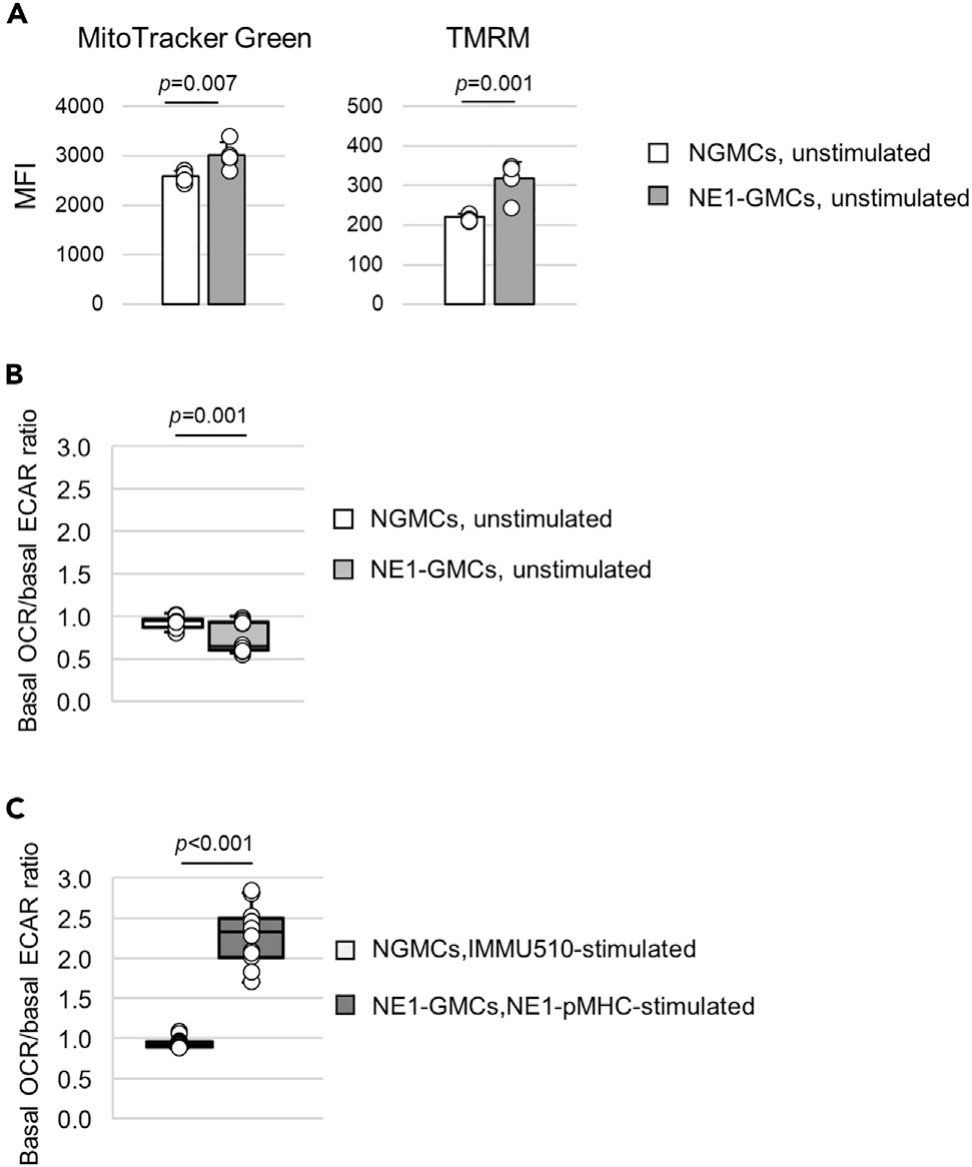
Mitochondrial phenotype assessment by flow cytometry using mitochondria-labeling dyes and extracellular flux analysis of NGMCs and NE1-GMCs (A) The mitochondrial phenotype of NGMCs and NE1-GMCs was assessed by flow cytometry using mitochondria-labeling dyes. The bar graph shows the average of five measurements of the mean fluorescence intensity (MFI) of MitoTracker Green and TMRM. (B) Boxplots (median with the 25th and 75th percentiles) of the basal OCR/basal ECAR ratio comparing unstimulated NGMCs (total 18 measurements from 2 experiments) and unstimulated CD8^+^ cells selected from NE1-GMCs (total 18 measurements from 3 experiments). The median basal OCR/basal ECAR ratios were 0.95 and 0.65, respectively. (C) Boxplots (median with the 25th and 75th percentiles) of the basal OCR/basal ECAR ratio of IMMU510-stimulated NGMCs (light gray, total 12 measurements from 2 experiments) and NE1-pMHC-stimulated CD8^+^ cells purified from NE1-GMCs (dark gray, total 15 measurements from 2 experiments). The median basal OCR/basal ECAR ratios were 0.92 and 2.33, respectively.

The mitochondrial phenotype of NGMCs and NE1-GMCs was assessed using MitoTracker Green and tetramethylrhodamine methyl ester (TMEM). NE1-GMCs had higher mitochondrial mass and mitochondrial membrane potential in the unstimulated state (Figure 5A). Another αβ-TCR-transduced GMCs, p40Tax-GMCs, also showed a similar mitochondrial phenotype (Figure S7A).

### Metabolic status analyzed by extracellular flux

Cellular metabolic status was assessed by extracellular flux analysis, measuring the oxygen consumption rate (OCR) and extracellular acidification rate (ECAR), with the results representing the levels of mitochondrial respiration and glycolysis, respectively. In the absence of TCR stimulation, the median basal OCR/basal ECAR ratio of NGMCs was similar to or higher than that of NE1-GMCs (NGMCs: 0.95 vs. NE1-GMCs: 0.65) (Figures 5B and S7B). With TCR stimulation, the median basal OCR/basal ECAR ratio increased only in NE1-GMCs, indicating enhanced mitochondrial metabolism in NE1-GMCs upon αβ-TCR stimulation (NGMCs: 0.92 vs. NE1-GMCs: 2.33) (Figure 5C). A similar change was observed in p40Tax-GMCs (Figure S7C).

## Discussion

In a study on TCR-T cells, an autoreactive TCR consisting of endogenous TCR and engineered TCR caused lethal toxicity in a mouse model.^14^ Considering the use of allogeneic γδ-T cells for αβ-TCR-transduced T cell therapy, T cell products would be safe because genetically engineered T cells are at least dual receptor-like, with monoclonal αβ-TCR and γδ-TCR. Acute rejection due to HLA incompatibility can be controlled by prior lymphodepleting chemotherapy. Therefore, if αβ-TCR-transduced γδ-T cells persist, the clinical development of off-the-shelf αβ-TCR-transduced allogeneic γδ-T cell products will advance greatly. The *in vivo* persistence of αβ-T cells is thought to be related to antitumor efficacy, and mainly in the context of CAR-T cell development, incorporation of 4-1BB (CD137) signaling^28^^,^^29^^,^^30^ and cytokines such as IL-7 and IL-15^31^ into T cells has been studied. Vγ9^+^Vδ2^+^ T cells are an innate-like T cell population.^32^ Clinical trials on these cells have not shown a remarkable survival benefit. The reason for this lack of efficacy is unclear^33^; however, their short persistence after activation may be a cause. In our study, αβ-TCR-transduced γδ-T cells showed promising persistence *in vitro* and *in vivo*. Lope et al. reported that IFN-γ secretion by γδ-T cells was glycolysis-dependent,^34^ which is consistent with our data. Mitochondrial function is useful for assessing the persistence of αβ-T cells^35^^,^^36^ and may be relevant for γδ-T cells. Here, we found that αβ-TCR-transduced γδ-T cells could utilize more mitochondrial energy metabolism.

In our study, αβ-TCR transduction increased the mitochondrial mass and mitochondrial membrane potential of γδ-T cells. αβ-TCR-transduced γδ-T cells contained more metabolites in the TCA cycle and OXPHOS and had higher gene expression of the mitochondrial electron transport chain in both unstimulated and stimulated states. In the stimulated state, IFN-γ production was more dependent on ATP synthesis, which was supported by the higher OXPHOS gene expression observed in αβ-TCR-transduced γδ-T cells. Although not high on the list in our gene analysis, our examinations using metabolic inhibitors suggest that energy through glutamine and fatty acid metabolism might also be important for the characteristics of αβ-TCR-transduced γδ-T cells. The metabolism of glutamine and fatty acids in αβ-TCR-transduced γδ-T cells requires further study.

Evaluation of spare respiratory capacity (SRC) is a useful method for assessing mitochondrial function in T cells.^37^^,^^38^ However, it was difficult to identify differences in SRC between TCR-stimulated NGMCs and NE1-GMCs. In contrast to SRC, the basal OCR/basal ECAR ratio can compensate for individual differences.^39^ An increased basal OCR/basal ECAR ratio was observed in NE1-pMHC-stimulated NE1-GMCs, which might reflect an inclination toward mitochondrial energy metabolism. Similar results were obtained for p40Tax-pMHC-stimulated p40Tax-GMCs. In a mouse model, a relationship between γδ-T cell subsets classified by cytokine secretion (IFN-γ or IL-17) and mitochondrial function was reported,^34^ but there were no obvious differences in cytokine profiles between NGMCs and NE1-GMCs induced from human donors in our study. The small sample size is one of the limitations of our study and requires further validation. However, our experiments consistently suggest that αβ-TCR-transduced γδ-T cells acquire superior mitochondrial function.

Recently, Stenger et al. reported the importance of the presence of αβ-TCR for αβ-T cell persistence by showing that knock out of endogenous TCR in CD19 CAR-T cells resulted in a lack of persistence, although the mechanism remains unclear.^40^ Mitochondria play an important role in the sustained killing of cytotoxic T cells.^41^ In our study, αβ-TCR and CD8 transduction to γδ-T cells yielded promising T cell persistence with higher utilization of mitochondrial function. CD8 is necessary to stabilize αβ-TCR recognition of the peptide epitope present on MHC molecules.^16^ The recruitment of CD8-associated Lck is essential for the augmentation of TCR signal transduction^42^ and *in vivo* persistence of αβ-T cells.^43^ These results support the enhanced antitumor activity of αβ-TCR and CD8 gene-cotransduced γδ-T cells. The interaction of αβ-TCR with nonstimulatory pMHC is known as coagonism.^44^^,^^45^^,^^46^^,^^47^ Appropriate Lck activation seems to be important for CD8^+^ T cell effector function and proliferation.

In conclusion, αβ-TCR transduction into γδ-T cells showed promising *in vitro* and *in vivo* persistence. As a mechanism, γδ-T cells could utilize more mitochondrial energy metabolism due to the acquisition of αβ-TCR and CD8.

### Limitations of the study

The study has several limitations: small sample size and reporting from a single group.

## STAR★Methods

### Key resources table

**Table:** 

REAGENT or RESOURCE	SOURCE	IDENTIFIER
**Antibodies**		

anti-human TCR αβ-biotin	Miltenyi Biotec	Cat#130-113-529
antibiotin microbeads	Miltenyi Biotec	Cat#130-090-485
CD8 microbeads	Miltenyi Biotec	Cat#130-045-201
Human TruStain FcX	BioLegend	Cat#422302
anti-human TCR Vδ2	BioLegend	Cat#331418, 331428, 331406
anti-human CD8a	BioLegend	Cat#301006, 301036
anti-human CD3ε	BioLegend	Cat#344815
anti-human CD45	BioLegend	Cat#304015
anti-human CD45RA	BD Biosciences	Cat#561216
anti-human CD27	BioLegend	Cat#302810
anti-human IFN-γ	BioLegend	Cat#502532
anti-Ki67	Beckman Coulter	Cat#B68180
TCRγδ-specific agonistic mAb	Beckman Coulter	Cat# PN IM1349

**Chemicals, peptides, and recombinant proteins**		

tetrakis-pivaloyloxymethyl 2-(thiazole-2-ylamino) ethylidene-1,1-bisphosphonate	Tanaka, Y. et al.^23^	N/A
RetroNectin	Takara Bio	Cat#T100A
GolgiStop Protein Transport Inhibitor	BD Biosciences	Cat#554724
Live or dead fixable dead cell staining kit NIR fluorescence	AAT Bioquest	Cat#22605
CytoTell Red 650	AAT Bioquest	Cat#22255
Apotracker Green	Biolegend	Cat#427402
2-deoxy-D-glucose	Sigma–Aldrich	Cat#D8375
Etomoxir	Cayman	Cat#11969
CB-839	Cayman	Cat#22038
Oligomycin	Abcam	Cat#141829
MitoTracker Green	Invitrogen	Cat#M7514
Tetramethylrhodamine (TMRM)	FUJIFILM	Cat#4987481499720

**Critical commercial assays**		

Combination of capillary electrophoresis and Fourier transform mass spectrometry (CE-FTMS)	Human Metabolome Technologies	N/A
Agilent RNA 6000 Pico Kit	Agilent Technologies	N/A
SurePrint G3 Human GE Microarray 8 × 60 K Ver3.0	Agilent Technologies	N/A
SureScan Microarray scanner	Agilent Technologies	N/A
Seahorse XF Cell Mito Stress Test Kit	Agilent Technologies	Cat#103010-100

**Deposited data**		

Raw RNA data	This paper	GEO: GSE216880

**Experimental models: Cell lines**		

NW-MEL-38	Memorial Sloan Kettering Cancer Center	RRID:CVCL_S591
T2	ATCC	RRID:CVCL_2211
SW982	ATCC	RRID:CVCL_1734
Fuji	ATCC	RRID:CVCL_D880

**Experimental models: Organisms/strains**		

NOD/Shi-*scid*/IL-2Rγ^null^	the Central Institute for Experimental Animals	N/A

**Software and algorithms**		

Gene set enrichment analysis (GSEA) version 4.3.2.	Broad Institute, Inc., Massachusetts Institute of Technology, and Regents of the University of California.	https://www.gsea-msigdb.org/gsea/index.jsp
SPSS Statistics version 26	IBM Japan	N/A

### Resource availability

#### Lead contact

Further information and requests for resources and reagents should be directed to and will be fulfilled by the lead contact, Mikiya Ishihara (mishihara@med.mie-u.ac.jp).

#### Materials availability

This study did not generate new unique reagents.

### Experimental models and study participants

#### Animals

NOD/Shi-*scid*/IL-2Rγ^null^ (NOG) female mice were purchased from the Central Institute for Experimental Animals (Kawasaki, Japan). Mice were fed a standard diet, housed under specific pathogen-free conditions, and used at 6–8 weeks of age. Five days after subcutaneous NW-MEL-38 melanoma cell inoculation, the mice were treated with total body irradiation (TBI) (2 Gy) and then infused with or without NE1-GMCs. The number of infused NE1-GMCs was 5×10^6^ cells/mouse. All animal experiments were conducted according to protocols approved by the Animal Care and Use Committee of the Mie University Life Science Center.

#### Cell lines

T2 cells (HLA-A2^+^, TAP-deficient, Epstein–Barr virus-transformed lymphoblastoid cell line), SW982 cells (NY-ESO-1^+^/HLA-A2^+^ synovial sarcoma cell line), Fuji cells (NY-ESO-1^-^/HLA-A2^+^ synovial sarcoma cell line), and NW-MEL-38 cells (HLA-A2^+^, NY-ESO-1^+^ melanoma cell line) were cultured in RPMI-1640 medium supplemented with 25 mM HEPES, 10% fetal calf serum (FCS), 2 mM glutamine, 100 U/mL penicillin, and 100 μg/mL streptomycin. Mycoplasma contamination of the cell lines was checked, but not detected.

### Method details

#### Vector construction and preparation of αβ-TCR-engineered γδ-T cells

αβ-TCR-engineered γδ-T cells were prepared as follows: Lymphocytes were isolated from the peripheral blood of healthy donors. None of the healthy donors had a history of an active autoimmune disease or malignancy. All samples were collected after obtaining written informed consent as part of a study approved by the Ethics Committee of Mie University Hospital. After negative selection using anti-human TCR αβ-biotin and antibiotin microbeads (Miltenyi Biotec, Bergisch Gladbach, Germany), TCR αβ-negative cells were cultured in modified Yssel’s medium with 10% human type AB serum and PTA (day 0).^26^ The culture medium was replaced with fresh medium containing IL-7 and IL-15 on day 1, and half of the medium was changed every 2–3 days. For GMCs, proliferating lymphocytes were infected with retroviral vectors on RetroNectin (Takara Bio Inc., Shiga, Japan)-coated plates on days 4 and 5. For NE1-GMCs, retroviral vectors encoding the G50A + A51E TCR (NE1_p157_/HLA-A∗02:01-specific TCR-α and TCR-β chains with G50A + A51E amino acid replacements) and human CD8 (Figure 1A) were used. An NY-ESO-1-specific TCR has been reported by Schmid et al.^10^ On day 8, γδ-T cells were harvested and frozen with CELLBANKER1 (Takara Bio) until use, except for metabolomic and transcriptome analyses. Each experiment was performed within two months of γδ-T-cell freezing. Thawed γδ-T cells were cultured in RPMI 1640 medium with 10% FCS for 4–6 h and then used in each experiment.

All procedures performed in this study involving human participants were conducted in accordance with the Japanese law of Ethical Guidelines for Medical and Biological Research Involving Human Subjects.

#### *In vitro functional* assay *based on flow cytometry*

Cytokine secretion and TCR expression in γδ-T cells were measured using flow cytometry. γδ-T cells were cultured with Fc Receptor Blocking Solution Human TruStain FcX (BioLegend) prior to antibody staining. Fluorescently labeled anti-human TCR Vδ2 monoclonal antibody (mAb) (Clone B6), anti-human CD8a mAb (clone RPA-T8), anti-human CD3ε mAb (clone SK7), anti-human CD45 mAb (clone HI30), anti-human IFN-γ mAb (clone 4S. B3) were purchased from BioLegend, Inc., and anti-Ki67 mAb was purchased from Beckman Coulter, Inc. (Brea, CA, USA). The live or dead fixable dead cell staining kit NIR fluorescence (AAT Bioquest, Inc., Sunnyvale, CA, USA) was used to identify the live cells. For T cell stimulation, γδ-T cells were cultured in PTA (1.0 μM)-containing medium on a TCRγδ-specific agonistic mAb (clone IMMU510) (Beckman Coulter) (1.0 μg/mL)-coated plate or NE1_p157_/HLA-A∗02:01 complex (NE1-pMHC) (1.0 ng/μL)-coated plate. For the intracellular staining of cytokines, γδ-T cells were cultured for 5–6 h with a GolgiStop Protein Transport Inhibitor (Nippon Becton Dickinson Company, Ltd., Tokyo, Japan). For metabolic inhibition experiments, RPMI 1640 (glucose-free) medium, 2-DG (Sigma–Aldrich Co. LLC, St. Louis, MO), etomoxir, CB-839 (Cayman Chemical, Ann Arbor, MI), and oligomycin (Abcam, Cambridge, UK) were used. For mitochondrial phenotype assessment, MitoTracker Green (Invitrogen) and tetramethylrhodamine (TMRM) (FUJIFILM) were used.

#### Metabolomic analysis

Comprehensive analysis of hydrophilic and ionic metabolites using a combination of capillary electrophoresis and Fourier transform mass spectrometry (CE-FTMS) was performed by Human Metabolome Technologies, Inc. (Yamagata, Japan). NE1-GMCs were purified using CD8 Microbeads (Miltenyi Biotec) on day seven. Metabolic extracts were prepared from 2.0-3.5 × 10^6^ cells per sample, with three samples from three different healthy donors included in each group, and methanol containing an internal standard solution after washing with mannitol-containing solution on day 8. Prior to extraction, T cells were cultured for 6 h in RPMI 1640-10% FCS medium with or without TCR stimulation. TCRγδ-specific agonistic mAb- and NE1-pMHC-coated plates were used for NGMCs and NE1-GMCs, respectively.

#### Transcriptomic analyses

RNA extracts were prepared from 3.1 × 10^5^ cells per sample from a healthy donor using a QIAamp RNA Blood Mini kit (QIAGEN K.K., Tokyo, Japan). Prior to RNA extraction, NGMCs and NE1-GMCs purified with CD8 microbeads were cultured with or without TCR stimulation, as in metabolomic analysis. RNA microarray analysis was performed by Agilent Technologies, Inc. Briefly, the RNA integrity of the samples was determined using an Agilent 2100 Bioanalyzer and Agilent RNA 6000 Pico Kit. All samples showed an RNA integrity number (RIN) higher than eight and qualified for further processing. Total RNA was reverse-transcribed to cDNA and then synthesized into Cy3-labeled cRNA by *in vitro* transcription. Labeled samples were hybridized to a SurePrint G3 Human GE Microarray 8 × 60 K Ver3.0. Scanning was performed using a SureScan Microarray scanner. Gene set enrichment analysis (GSEA)^48^ was performed using GSEA software version 4.3.2. (https://www.gsea-msigdb.org/gsea/index.jsp). The log2-transformed, normalized and validated gene data by Agilent Technologies, Inc. was used. The genes were ranked based on the gene set ‘WikiPathways subset of Canonical Pathways’.

#### Measurement of the oxygen consumption rate (OCR) and extracellular acidification rate (ECAR)

OCR and ECAR were measured with a Seahorse XF HS Mini Analyzer using a Seahorse XF Cell Mito Stress Test Kit (Agilent Technologies Inc., Santa Clara, CA, USA). Briefly, after thawing, γδ-T cells were cultured in RPMI 1640-10% FCS medium. The TCR stimulation time was 72 h. After washing with assay medium, γδ-T cells were plated at 2.0–3.0 × 10^5^ cells/well, with the same number of cells included in each experiment, in a cell culture plate with assay medium and cultured for a total of 50–60 min at 37°C in a CO_2_-free incubator until analysis. The final concentrations of oligomycin, FCCP, and rotenone/antimycin A were 1.5, 1.0 μM and 0.5 μM, respectively. The basal OCR/basal ECAR ratio was calculated using the equation: (pmol/min)/(mpH/min).^39^

### Quantification and statistical analysis

For cytokine inhibition studies, a t test was used if Levene’s test showed a value above the significance level; otherwise, Welch’s t test was used. For metabolic status analysis using Seahorse, the Mann–Whitney U test was used. Calculations were performed using SPSS Statistics version 26 (IBM Japan, Ltd., Tokyo, Japan). For the metabolomic analysis, we described the results of Welch’s test reported by Human Metabolome Technologies, Inc. p values less than 0.05 were considered statistically significant (∗p < 0.05, ∗∗p < 0.01).

## Data Availability

• The RNA data generated in this study have been deposited at Gene Expression Omnibus (GEO) and are publicly available as of the date of publication. • Accession number is listed in the key resources table. • Any additional information required to reanalyze the data reported in this paper is available from the lead contact upon request.
