# Supplementary material for: αβ-T cell receptor transduction gives superior mitochondrial function to γδ-T cells with promising persistence
Source: iScience. 2023 Aug 31;26(10):107802. doi: 10.1016/j.isci.2023.107802 (PMC10502403; doi:10.1016/j.isci.2023.107802)
Supplement: Document S1. Figures S1–S7 and Tables S1 and S2 [file mmc1.pdf]

iScience, Volume *26*

Supplemental information

αβ-T cell receptor transduction gives superior mitochondrial function to γδ-T cells with promising persistence

Mikiya Ishihara, Hiroshi Miwa, Hiroshi Fujiwara, Yasushi Akahori, Takuma Kato, Yoshimasa Tanaka, Isao Tawara, and Hiroshi Shiku

Supplementary Table S1. Frequency of IFN-γ-secreting cells with metabolic inhibitors, related to Figure 2.

NGMCs (Vδ2^{+}), IFN-γ-secreting cells (%)

	Unstimulated	IMMU510-stimulated	IMMU510 stimulation +			
			2-DG	Etomoxir	CB-839	Oligomycin
1	0.1	76.1	32.9	35.6	34.7	42.5
2	1.1	80.9	38.1	42.1	34.6	35.2
3	0.8	76.6	32.0	36.9	34.0	31.1
4	0.0	90.6	53.4	83.3	83.5	71.1
5	0.0	89.1	45.0	60.0	47.7	54.9
6	0.1	85.7	52.4	62.4	62.7	64.1

Using γδ-T cells induced from three healthy donors.

NE1-GMCs (Vδ2^{low}CD8^{high}), IFN-γ-secreting cells (%)

	Unstimulated	NE1-pMHC-stimulated	NE1-pMHC stimulation +			
			2-DG	Etomoxir	CB-839	Oligomycin
1	0.1	76.5	23.1	29.4	37.3	13.6
2	0.5	72.4	13.9	34.7	33.8	21.3
3	0.3	77.2	18.1	24.1	23.0	17.2
4	0.0	96.1	77.8	61.8	60.2	65.2
5	0.0	41.6	26.4	11.7	11.7	8.7

Using γδ-T cells induced from three healthy donors.

NE1-GMCs, genetically modified γδ-T cells expressing an NY-ESO-1-specific αβ-TCR and the CD8 coreceptor; NE1-pMHC, NY-ESO-1p$_{157-165}$ peptide and HLA-A*02:01 complex; NGMCs, nongene-modified γδ-T cells.

Supplementary Table S2. Main correlation factors of metabolic pathways, related to Figure 3.

Pathway	Metabolite	Comparative Analysis						Correlation factor	
		NE1-GMCs, unstimulated vs NGMCs, unstimulated			NE1-GMCs, NE1-pMHC-stimulated vs NGMCs, IMMU510-stimulated			PC1	PC2
		Ratio	*p*-value		Ratio	*p*-value			
Glycolysis	Glucose 6-phosphate	1.0	0.670		1.0	0.917		3.9E-02	9.3E-01
	Fructose 6-phosphate	1.1	0.419		1.3	0.400		1.6E-01	9.6E-01
	Glyceraldehyde 3-phosphate	<1	N.A.		0.5	0.037	*	-1.9E-01	8.0E-01
	3-Phosphoglyceric acid	1.1	0.800		0.9	0.749		2.9E-01	7.4E-01
	Phosphoenolpyruvic acid	0.8	N.A.		0.8	0.582		1.3E-01	7.9E-01
	Acetyl CoA_divalent	1.4	N.A.		0.3	0.082		-3.0E-01	3.7E-01
	CoA_divalent	1.3	0.235		1.3	0.641		6.5E-01	5.6E-01
TCA cycle and OXPHOS	Citric acid	1.8	0.144		1.4	0.229		8.3E-01	-4.7E-01
	cis-Aconitic acid	1.8	0.117		1.3	0.240		7.7E-01	-5.6E-01
	2-Oxoglutaric acid	1.8	0.251		1.5	0.289		7.3E-01	-5.0E-01
	Succinic acid	1.7	0.333		1.2	0.482		8.6E-01	-2.1E-01
	Fumaric acid	1.7	0.255		1.4	0.471		9.0E-01	1.4E-01
	Malic acid	1.8	0.211		1.7	0.344		9.1E-01	7.0E-02
	NADH	1.5	0.288		1.6	0.285		9.1E-01	-2.2E-01
	NAD^+	1.5	0.051		1.5	0.310		8.2E-01	-4.8E-01
	ADP	1.3	0.297		1.2	0.472		8.6E-01	-4.2E-01
	ATP	1.2	0.269		1.3	0.307		5.3E-01	-6.7E-01
Others	cAMP	0.6	0.468		1.4	N.A.		1.1E-01	-6.6E-01
	AMP	1.2	0.660		1.0	0.951		7.8E-01	2.4E-02
	Glutamine	1.3	0.344		1.2	0.171		3.1E-01	9.2E-01
	Glutamic acid	1.3	0.252		1.3	0.180		8.9E-01	-3.0E-01
	Glutathione (GSH)	1.8	0.073		1.7	0.441		8.1E-01	-3.2E-01
	Glutathione (GSSG)_divalent	1.3	0.397		1.5	0.007	**	6.4E-01	-5.4E-01
	HMG CoA_divalent	2.3	0.465		1.3	0.506		7.2E-01	3.2E-01
	3-Hydroxybutyric acid	1.6	0.017	*	1.5	0.069		7.7E-01	-3.0E-01
	Arg	1.2	0.362		1.4	0.177		2.3E-01	9.5E-01
	Ornithine	1.1	0.690		1.2	0.324		5.6E-02	9.4E-01
	Citrulline	1.3	0.046	*	1.2	0.478		5.3E-01	7.4E-01
	Betaine	1.7	0.073		1.6	0.015	*	6.7E-01	-2.5E-01
	IMP	1.0	0.819		1.0	0.939		-1.8E-01	8.0E-01

Comparative analysis in this table was analysed using Welch's t test. *p<0.05, **p<0.01.

NE1-GMCs, genetically modified γδ-T cells expressing an NY-ESO-1-specific αβ-TCR and the CD8 coreceptor; NE1-pMHC, NY-ESO-1p$_{157-165}$ peptide and HLA-A*02:01 complex; NGMCs, nongene-modified γδ-T cells.

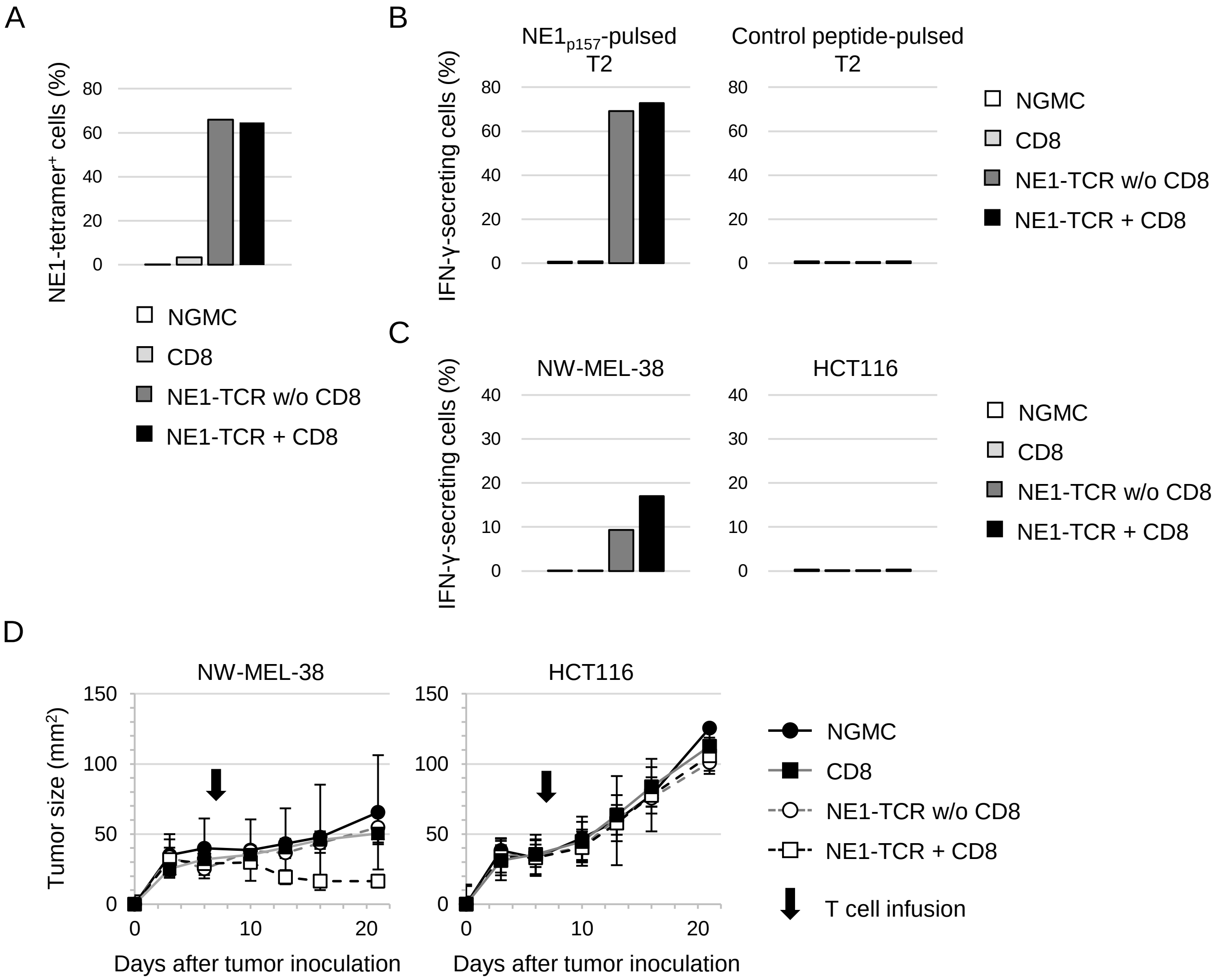

Supplementary Figure S1: Coexpression of CD8 enhanced the antitumor effect of αβ-TCR-transduced γδ-T cells, related to Figure 1.

(A) Frequencies of NY-ESO-1 tetramer (NE1 tetramer)-positive cells in nongene-modified γδ-T cells (NGMCs), γδ-T cells genetically modified with a CD8αβ gene-encoding retroviral vector (CD8-GMCs), γδ-T cells genetically modified with a G50A+A51E αβ-TCR gene-encoding retroviral vector (NE1-TCR w/o CD8-GMCs), and γδ-T cells genetically modified with the G50A+A51E αβ-TCR gene-encoding retroviral vector and CD8αβ gene-encoding retroviral vector (NE1-TCR + CD8-GMCs). (B-C) The frequencies of IFN-γ-secreting cells following stimulation with peptide-pulsed T2 cells (B) and the NW-MEL-38 melanoma cell line (HLA-A2$^+$, NY-ESO-1$^+$) or HCT116 colon cancer cell line (HLA-A2$^+$, NY-ESO-1$^-$) for NGMCs, CD8-GMCs, NE1-TCR w/o CD8-GMCs, and NE1-TCR + CD8-GMCs. (D) Tumor size change: NW-MEL-38 or HCT116 tumor cells were inoculated subcutaneously into the back of NOG mice. A total of 1×10^7 cells were infused 7 days after tumor inoculation (black arrow). The number of assessable mice was 2 for the NGMC group and 3 for the CD8-GMC, NE1-TCR w/o CD8-GMC and NE1-TCR + CD8-GMC groups. Each bar indicates the standard deviation.

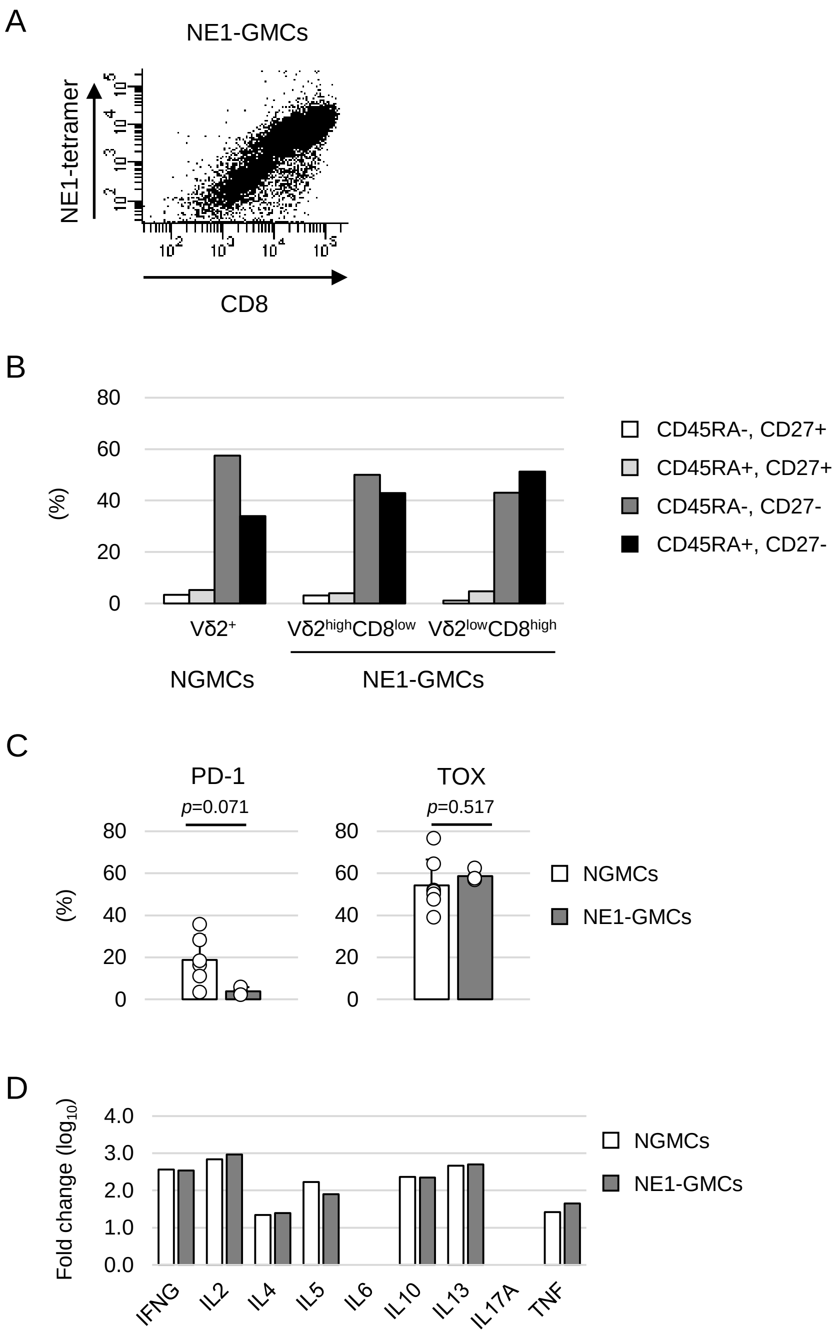

Supplementary Figure S2: Characteristics of NGMCs and NE1-GMCs, related to Figure 1. (A) Representative data of expression of the CD8 and a tetramer recognizing the NY-ESO-1-specific TCR (NE1-tetramer) in NE1-GMCs. (B) Representative data on the immunophenotype of NGMCs (left), $V\delta2^{high}CD8^{low}$ NE1-GMCs (middle) and $V\delta2^{low}CD8^{high}$ NE1-GMCs (right). (C) PD-1 and TOX expression in NGMCs and $V\delta2^{low}CD8^{high}$ NE1-GMCs. (D) Fold change in the RNA expression of cytokines in NGMCs and NE1-GMCs purified using CD8 Microbeads after TCR stimulation. RNA microarray analysis was performed by Agilent Technologies, Inc. IMMU510 was used for γδ-TCR stimulation, and NE1-pMHC was used for NE1-TCR stimulation.

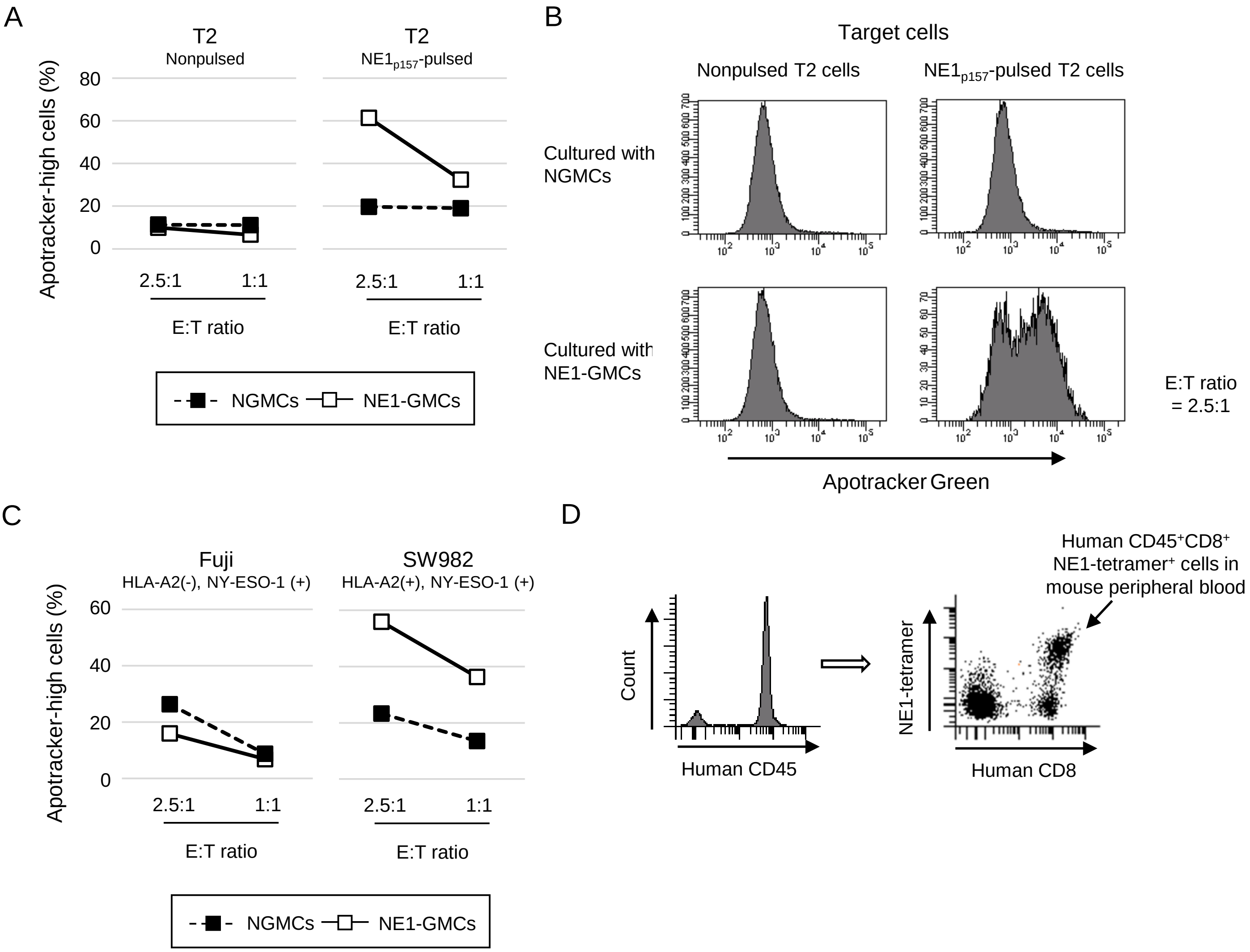

Supplementary Figure S3: Flow cytometric apoptosis assay using Apotracker Green, related to Figure 1. (A) Apoptosis assay performed with peptide-pulsed cells. NGMCs (black square) or NE1-GMCs (white square) were cultured with nonpulsed T2 cells (left) or NY-ESO-$1_{p157\text{-}165}$ peptide ($NE1_{p157}$)-pulsed T2 cells (right). The effector-to-target cell (E:T) ratios were 2.5:1 and 1:1. (B) Histogram of the Apotracker Green signal in target cells is shown. Effector cells (NGMCs (upper panel) or NE1-GMCs (lower panel)) were cultured with target cells (nonpulsed T2 cells (left) or NY-ESO-$1_{p157\text{-}165}$ peptide ($NE1_{p157}$)-pulsed T2 cells (right)). The effector-to-target cell (E:T) ratio was 2.5:1. (C) Apoptosis assay performed with NY-ESO-1^{+} tumor cells. NGMCs (black square) and NE1-GMCs (white square) were cultured with target sarcoma cells, Fuji cells (HLA-$A2^{-}$, NY-ESO-1^{+}, left) and SW982 cells (HLA-$A2^{+}$, NY-ESO-1^{+}, right). The E:T ratios were 2.5:1 and 1:1. (D) Representative flow cytometry data obtained from the NE1-GMC-infused mouse in Figure 2A.

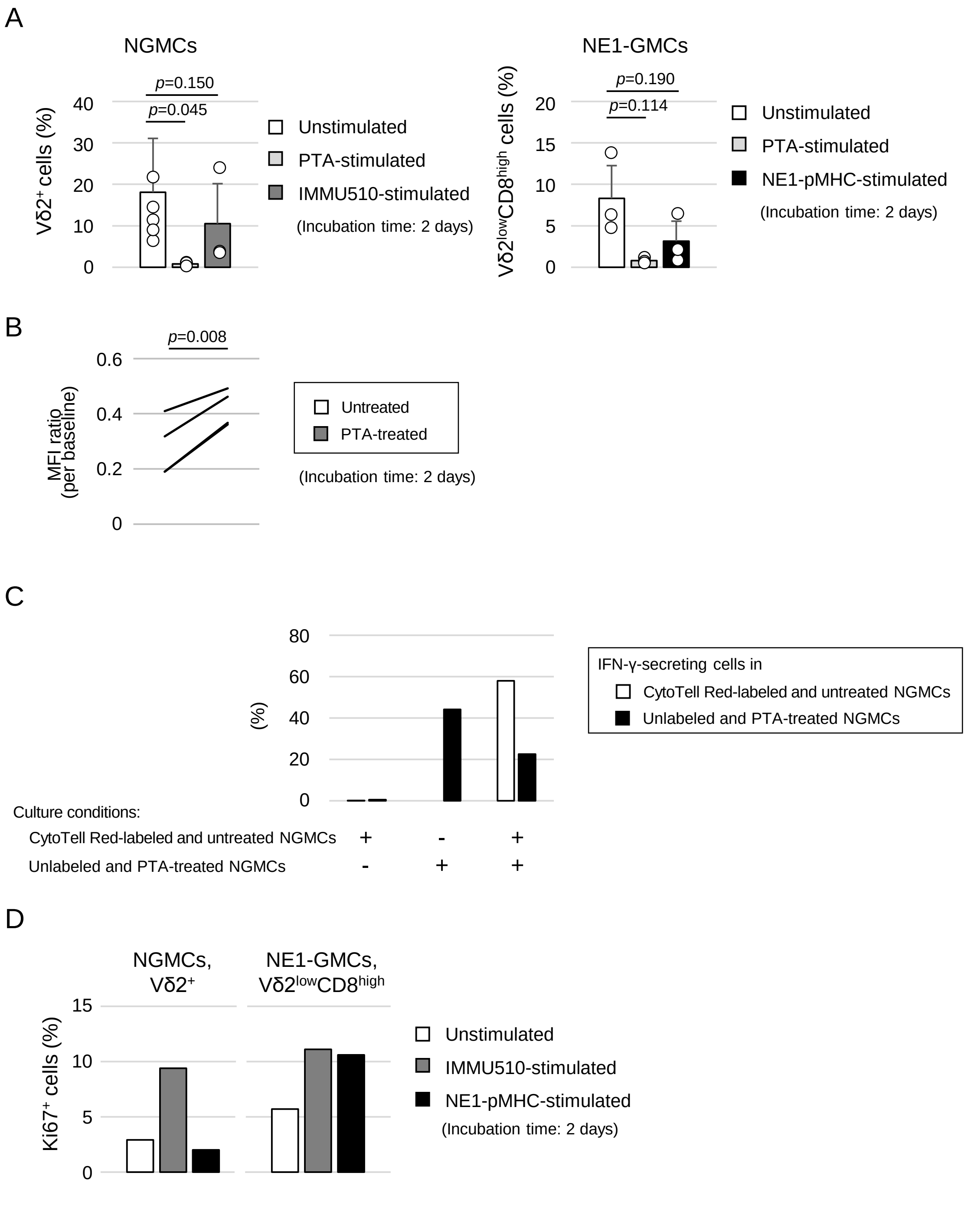

Supplementary Figure S4: PTA suppressed γδ-T-cell growth, related to Figures 1 and 2.
(A) Frequencies of NGMC Vδ2$^+$ cells and NE1-GMC Vδ2^{low}CD8^{high} cells 2 days after stimulation. Cytokines were not added to the culture medium. The frequencies of cells were calculated as follows: (Vδ2$^+$ cells per NGMCs, or Vδ2^{low}CD8^{high} cells per NE1-GMCs) × (gated lymphocytes per all events). The error bar shows the standard deviation. (B) Mean fluorescence ratio of CytoTell Red-labeled NGMCs. The ratio of MFI at 2 days to that at baseline from 4 experiments is shown. Boxplots (median with the 25th and 75th percentiles) of the mean MFI ratio of untreated and PTA-treated NGMCs (n=4). Each line connects the pair. (C) IFN-γ secretion by NGMCs was evaluated by intracellular staining. Untreated NGMCs were labeled with CytoTell Red. Untreated NGMCs secreted IFN-γ when cultured with PTA-treated NGMCs. (D) Frequency of Ki67$^+$ cells in NGMC Vδ2$^+$ cells and NE1-GMC Vδ2^{low}CD8^{high} cells, with or without stimulation. Cytokines were not added to the culture medium. Another experiment showed similar results.

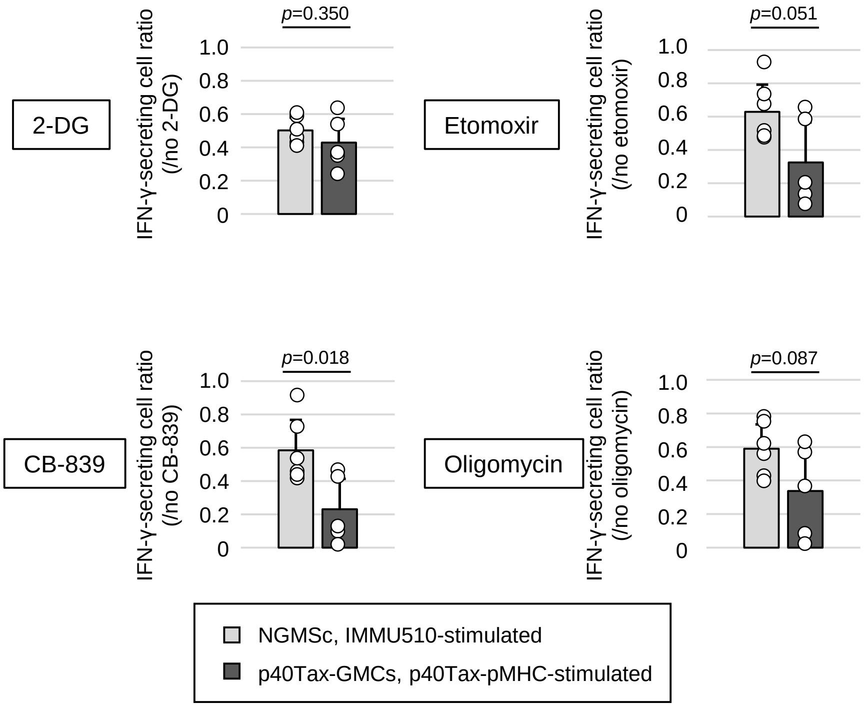

Supplementary Figure S5: Metabolism dependencies of IFN-γ secretion by p40Tax-GMCs, related to Figure 2.

Frequencies of IFN-γ-secreting cell ratios determined by comparing PTA or IMMU510-stimulated NGMC Vδ2^{+} cells (n=6) and p40Tax-pMHC-stimulated p40Tax-GMC Vδ2^{low}CD8^{high} cells (n=5). The concentrations of metabolic inhibitors were 2-DG (10 mM), etomoxir (100 μM), CB-839 (2 μM), or oligomycin (2 μg/mL). The error bar shows the standard deviation.

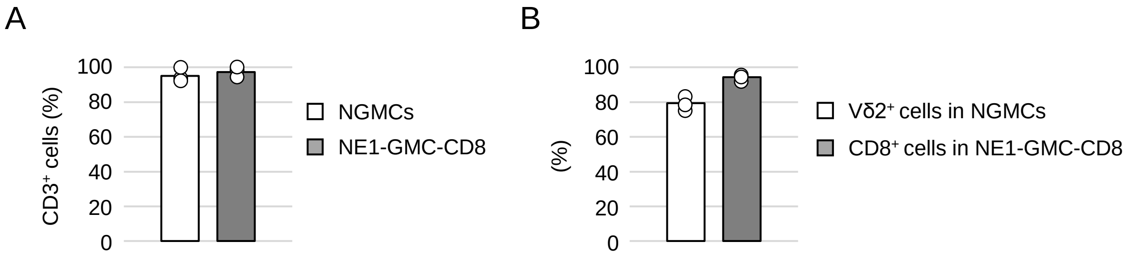

Supplementary Figure S6: Characteristics of NGMCs and NE1-GMCs used for metabolomic analysis, related to Figure 3.

γδ-T cells (day 8, without freezing and thawing) induced from 3 different healthy donors were used for metabolomic analysis. NE1-GMCs were purified with CD8 Microbeads® (NE1-GMC-CD8). (A) Mean frequency of $CD3^+$ cells. (B) Mean frequency of $V\delta 2^+$ cells in NGMCs (left) and $CD8^+$ cells in NE1-GMC-CD8 (right). Error bars indicate standard deviation.

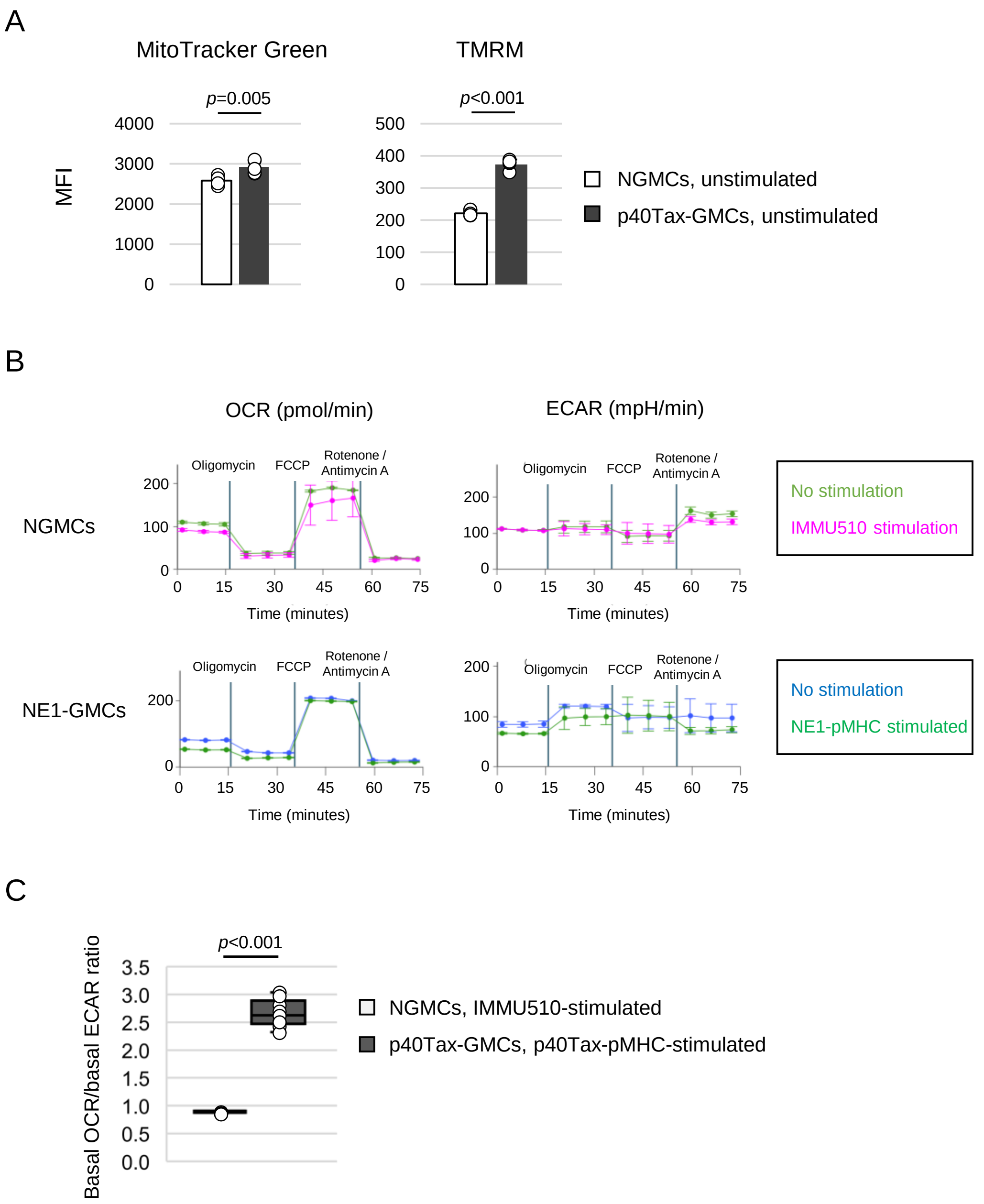

Supplementary Figure S7: Mitochondrial functional assessment using flow cytometry and a Seahorse XF HS Mini Analyzer, related to Figures 4 and 5.
(A) MFI of MitoTracker® Green and tetramethylrhodamine, methyl ester (TMEM) staining of NGMCs Vδ2^{+} cells and p40Tax-specific αβTCR and CD8αβ-transduced GMCs (p40Tax-GMCs) Vδ2^{low}CD8^{high} cells in an unstimulated state are shown. The bar graph shows the average MFI of five measurements. The t test was used. (B) Representative data of the OCR and ECAR of NGMCs and NE1-GMCs are shown. (C) Boxplots (median with the 25th and 75th percentiles) of the basal OCR/basal ECAR ratio comparing IMMU510-stimulated NGMCs (6 measurements using γδ-T cells induced from 1 healthy donor) and p40Tax-pMHC-stimulated CD8^{+} cells selected from p40Tax-GMCs (9 measurements using γδ-T cells induced from 1 healthy donor). The median basal OCR/basal ECAR ratios were 0.90 and 2.62, respectively.
